# Detection of aberrant hippocampal mossy fiber connections: Ex vivo mesoscale diffusion MRI and microtractography with histological validation in a patient with uncontrolled temporal lobe epilepsy

**DOI:** 10.1002/hbm.23066

**Published:** 2015-11-27

**Authors:** Michel Modo, T. Kevin Hitchens, Jessie R. Liu, R. Mark Richardson

**Affiliations:** ^1^ Department of Radiology University of Pittsburgh Pittsburgh Pennsylvania; ^2^ Department of Bioengineering University of Pittsburgh Pittsburgh Pennsylvania; ^3^ McGowan Institute for Regenerative Medicine, University of Pittsburgh Pittsburgh Pennsylvania; ^4^ Centre for the Neural Basis of Behavior Pittsburgh Pennsylvania; ^5^ Department of Neurobiology University of Pittsburgh Pittsburgh Pennsylvania; ^6^ Department of Neurological Surgery University of Pittsburgh Pittsburgh Pennsylvania

**Keywords:** intractable epilepsy, surgical resection, ex vivo MRI, ultra high field MRI, diffusion tensor imaging, MR histology, cytoarchitecture, tractography, mossy fibers, sprouting hypothesis

## Abstract

Understanding the neurobiology and functional connectivity of hippocampal structures is essential for improving the treatment of mesial temporal lobe epilepsy. At the macroscale, in vivo MRI often reveals hippocampal atrophy and decreased fractional anisotropy, whereas at the microscopic scale, there frequently is evidence of neuronal loss and gliosis. Mossy fiber sprouting in the dentate gyrus (DG), with evidence of glutamatergic synapses in the stratum moleculare (SM) putatively originating from granule cell neurons, may also be observed. This aberrant connection between the DG and SM could produce a reverberant excitatory circuit. However, this hypothesis cannot easily be evaluated using macroscopic or microscopic techniques. We here demonstrate that the ex vivo mesoscopic MRI of surgically excised hippocampi can bridge the explanatory and analytical gap between the macro‐ and microscopic scale. Specifically, diffusion‐ and T_2_‐weighted MRI can be integrated to visualize a cytoarchitecture that is akin to immunohistochemistry. An appropriate spatial resolution to discern individual cell layers can then be established. Processing of diffusion tensor images using tractography detects extra‐ and intrahippocampal connections, hence providing a unique systems view of the hippocampus and its connected regions. Here, this approach suggests that there is indeed an aberrant connection between the DG and SM, supporting the sprouting hypothesis of a reverberant excitatory network. Mesoscopic ex vivo MR imaging hence provides an exciting new avenue to study hippocampi from treatment‐resistant patients and allows exploration of existing hypotheses, as well as the development of new treatment strategies based on these novel insights. *Hum Brain Mapp 37:780–795, 2016*. © **2015 Wiley Periodicals, Inc.**

## INTRODUCTION

Mesial temporal sclerosis (MTS) is the most recognized finding in drug resistant chronic temporal lobe epilepsy (TLE) [Thom, 2014]. Based on the differential extent of neuronal loss in the Cornu Ammonis (CA) cell layers, it is possible to distinguish different patterns of MTS [Blumcke et al., 2012]. Although there are clear neuropsychological consequences of pyramidal cell loss, its significance in terms of etiology and phenomenology of seizures remains a matter of debate [Bonilha et al., 2012; Coras et al., 2014]. Nevertheless, the presence of MTS and the resultant tissue atrophy are key selection criteria for resective surgery, as patients with atypical or no MTS are less likely to achieve seizure freedom following resection [Bonilha et al., 2012].

Although extra‐hippocampal signaling might be an indirect contributor to aberrant signal transmission within the hippocampus [Sisodiya et al., 1997], a leading hypothesis is that granule cell axons (Mossy fibers) from a dispersing dentate gyrus (DG) form aberrant connections with neurons in the Stratum Moleculare, hence producing a reverberant excitatory circuit that results in seizure activity [Blumcke et al., 2009; Houser et al., 2012; O'Dell et al., 2012; Sharma et al., 2007]. Excitatory glutamatergic axons in the inner molecular layer are evident [Frotscher et al., 2006; Proper et al., 2000; Sutula and Dudek, 2007], but currently there is no direct evidence of these aberrant connections originating from the DG in human patients. Current histological and diagnostic techniques are insufficient to reveal and map these intrahippocampal connections [Bertram, 2009; Bonilha et al., 2012; Thom et al., 2010].

Understanding the seizure network, rather than merely the ictal focus (where aberrant neural activity converges to produce uncontrolled activity), will be an important advance to improve surgical interventions and care [Bertram, 2009; Kucukyuruk et al., 2012]. Investigating cellular, as well as network changes, in the human hippocampus and related areas is extremely challenging. With regard to available techniques for evaluating the human brain, standard neuropathological investigations using histochemistry and immunohistochemistry (IHC) afford a detailed analysis of the cellular composition of the tissue (microscale), while electron microscopy (EM) visualizes the ultrastructural changes of a few cells (nanoscale). As these techniques require sectioning, i.e. destruction of the excised hippocampus, reconstructing these data to reflect a three‐dimensional (3D) systems view of the whole hippocampus remains unsatisfactory and there is an ongoing effort to improve these approaches [Goubran et al., 2013; Tsuriel et al., 2015]. Often these studies hence focus on a very limited field of view, such as a few slices of the anterior hippocampus [O'Dell et al., 2012; Thom, 2014]. Using these techniques, it will be extremely challenging to identify pathways between different components of a network that provide the substrate for a functional connection (i.e. functional anatomy). Nevertheless, recent developments in optical coherence tomography (OCT) and polarized light imaging (PLI) are promising to extend the visualization of connectivity from the microscale to the mesoscale using optical techniques [Axer et al., 2011; Wang et al., 2014].

In contrast, non‐invasive approaches, such as MRI‐based diffusion tensor imaging (DTI) and electroencephalogram (EEG), allow whole brain (i.e. macroscale) investigations of anatomical and functional connectivity. Still, in patients, these two techniques lack the spatial resolution to interrogate networks at the intrahippocampal scale (∼5 mm diameter), with a DTI resolution of 2 × 2 × 2 mm (=8 μL) at 3 T and state‐of‐the‐art 0.7 mm in plane resolution at 7 T for each voxel (i.e. three to nine voxels per hippocampal diameter) [Concha et al., 2010; Goubran et al., 2014; Heidemann et al., 2010; Polders et al., 2011]. “High‐resolution” in vivo T_1_‐ or T_2_‐weighted MRI (achieving 0.5 mm in‐plane, 1 mm/slice) afford the distinction and potentially automated assignment of individual CA regions, the DG, and subiculum to provide measures of atrophy [Howe et al., 2010; Kerchner et al., 2010; Prudent et al., 2010; Schoene‐Bake et al., 2014a; Wisse et al., 2012]. However, considering that the CA1 field is merely 0.15 to 0.2 mm across, limitations, such as partial volume effects, raise concerns regarding how reliable these measurements can be, especially considering the absence of histological validation in these studies.

Several studies have performed high resolution images of postmortem human hippocampus [Adler et al., 2014; Chakeres et al., 2005; Yushkevich et al., 2009], with an in‐plane spatial resolution of 0.07 mm and a greater than 0.3 mm slice thickness [Shepherd et al., 2007]. Tractography has been used to visualize the perforant path (0.3 mm resolution) in ex vivo hippocampi [Augustinack et al., 2010, 2013], but so far has not revealed intrahippocampal connections or their implication in mTLE. Ideally, images with isotropic voxels are used for tractography at a resolution where intrahippocampal structures are clearly discernable. Macroscopic techniques may therefore be informative in understanding the macroscale (mm to cm) consequences of epilepsy, but current approaches are still insufficient to provide the detail required to map epileptogenic abnormalities in the network. Consequently, the mesoscale approach (μm to mm) needs to be further developed to eventually map a seizure network responsible for mTLE. Subsequently, a re‐interpretation of macroscale diffusion characteristics at 7 T based on downsampled mesoscale images will refine the diagnosis and surgical planning of patients with mTLE.

It is increasingly recognized that epilepsy is not a disorder restricted to the ictal focus; neuromodulation through input regions also plays an important role. Nevertheless, current investigative approaches (macroscale DTI and microscale IHC) are inadequate to investigate regional networks [Oh et al., 2014]. Dell'acqua et al. [2013] proposed an innovative strategy that bridges this gap of analytical approaches by applying mesoscale MRI‐based histology and microtractography. A major advantage of this approach is a more adequate assessment of microstructural diffusion effects within an imaging voxel, which reduces the potential partial volume effects present in macroscopic size voxels [Besseling et al., 2012]. Although tract tracing and network analysis is feasible in animal models of mTLE, species differences in structural and functional anatomy, as well as the specific methods of seizure induction, limit their validity to explain human mTLE [Insausti, 1993]. The approach outlined here will therefore make a unique contribution to improve our understanding of mTLE.

## MATERIALS AND EQUIPMENT

### Patient Selection

The subject was a 23‐year‐old man with pharmacologically intractable epilepsy recommended for anterior temporal lobectomy by an interdisciplinary epilepsy surgery board. The patient's seizure onset began at the age of 4 years, following a history of congenital toxoplasmosis infection. His preoperative 3 T MRI demonstrated subtle T2 hyperintensity and minimal volume loss in the right hippocampus, consistent with mesial temporal sclerosis. En bloc hippocampectomy was performed as standard surgical care. The specimen was collected as part of the Surgical Epilepsy Brain and Biomarker Databank at the University of Pittsburgh, under a protocol approved by the Institutional Review Board. Clinical histopathological evaluation revealed hippocampal sclerosis with selective CA3 neuronal loss.

### Specimen Preparation

**Figure 1:**
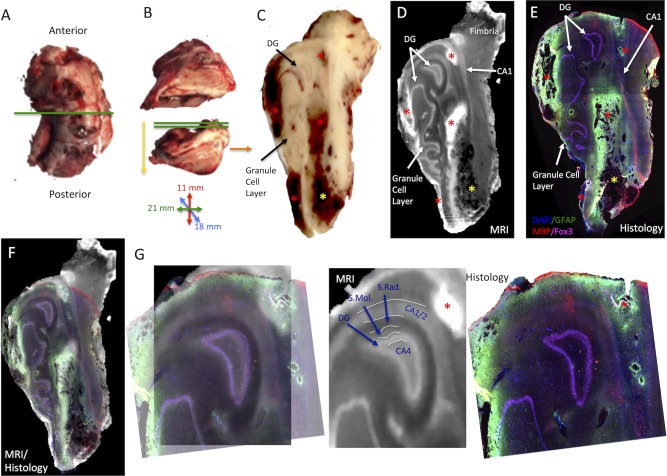
MR‐histology of the epileptic human hippocampus. **A**. The excised right hippocampus of a 23 years old man with intractable mTLE. **B**. The anterior portion is used for clinical pathology and the posterior part is available for research (green lines indicate orientation for histological sectioning). **C.** Blockface image of the HC before sectioning. **D**. The ADC image affords the detection of the DG and pyramidal cell layer. **E**. These features correspond to histological images visualizing cell nuclei (DAPI), astrocytes (GFAP), myelin basic protein (MBP), as well as neurons (Fox3). **F**. A combined MRI‐histology image highlights the overlap between both methods. **G**. A focus on the hippocampus indicates that the ADC image affords a distinction of CA4, DG, stratum moleculare (S.Mol.), s. radiatum (S.Rad.), and CA1/2, in accordance with fluorescence histology. [Color figure can be viewed in the online issue, which is available at http://wileyonlinelibrary.com.]

The excised hippocampus was cut in the coronal plane, the anterior portion sent for clinical neuropathological analyses, and the posterior portion was used for research (Fig. 1A,B). The specimen was transferred into 4% paraformaldehyde and postfixed for 48 h before being transferred to phosphate buffered saline (PBS) for storage at 4°C. Approximate dimensions of the research specimen were 11 × 21 × 18 mm. The coronal face of the posterior part of the hippocampus revealed the dentate gyrus, the pyramidal cell layer, as well as surgical trauma (Fig. 1C). For MR scanning (<1 month postexcision), the specimen was immersed in the proton‐free FluorInert (Sigma‐Aldrich, St. Louis MO), while avoiding the generation of air bubbles in a syringe that afforded immobilization of the specimen for the duration of the long scan time.

### MRI Scanning

Images were collected using an 11.7 T/89 mm Bruker Avance DBX microimaging scanner equipped with a Micro2.5 gradient set capable of 100 G/cm, a 20 mm diameter birdcage RF coil and Paravision 4.0 (Bruker Biospin, Billerica, MA). The sample was maintained at 4**°**C throughout scanning. A 3D T_2_‐weighted Spin Echo image series was collected with 16 equally spaced echoes (TR = 4,000 ms, base TE = 10 ms, NA = 1, FOV= 25.6 × 12.8 × 12.8 mm, matrix = 256 × 128 × 128, isotropic resolution of 100 μm) to provide high‐resolution anatomical reference images. A 3D Pulsed Gradient Spin Echo sequence was used to collect three DTI data sets (TR = 1,100 ms; TE = 28 ms; diffusion durations *δ* = 2.5 ms, 4 ms, 6.5 ms; spacing between diffusion gradients Δ = 15 ms; *b*‐value 4,000 s/mm^2^; NA = 1; 6 noncolinear diffusion directions; one A0 image; FOV = 25.6 × 12.8 × 12.8 mm; matrix 256 × 128 × 128; total number of voxels = 4,194,304; 100 μm isotropic resolution). To vary diffusion time, as well as G, diffusion duration was adjusted, while the *b*‐value was kept constant (Table 1). Variation of diffusion time has been shown to potentially target different length scales that could improve visualization of the tissue microstructure [Baron and Beaulieu, 2014; Van et al., 2014] and hence provide a better characterization of pathology in the living brain. Total scanning time was 124 h.

**Table 1:** Acquisition parameters used for diffusion tensor imaging

Scan	Duration, delta (*δ*)	Spacing, delta (Δ)	Diffusion time, Δ‐δ/3	Diffusion‐weighting, *b*‐value	Gradient strength, *G*
1	2.5 ms	15 ms	14.16 ms	4000 s/mm^2^	867 mT/m
2	4.0 ms		13.60 ms		544 mT/m
3	6.5 ms		12.80 ms		342 mT/m

### Processing of Diffusion Images

Diffusion images were processed using DSI studio [Yeh et al., 2013; http://dsi-studio.labsolver.org]. Data was preprocessed to correct eddy current distortions, as well as to mask the sample for further analyses. Fractional anisotropy (FA), apparent diffusion coefficient (ADC), radial diffusivity (RD), and axial diffusivity (AD) maps were generated and saved as NIFTI‐files. Pseudo‐color‐coding (Blue, Green, Red) of NIFTI files was performed in FIJI before creating overlays.

### Signal Intensity Comparisons

A key measure to determine if sufficient signal was acquired to distinguish the sample from background is the signal‐to‐noise ratio (SNR). For this, the signal (*S*) was defined as the mean voxel intensity value of a region of interest (ROI) over tissue. Noise (*N*) was calculated as the standard deviation (*σ*) in voxel intensity of an ROI in non‐tissue background free of any artifacts. SNR was hence calculated as *S*/*σN*. Although a SNR of at least 3:1 is recommended for tractography studies using a single receive coil [Jones et al., 2013], a safe minimum is assumed to be a 10:1 ratio [Descoteaux et al., 2009] with simple diffusion models (as in the case of six directions). Still, SNR values or methods for their calculation are not commonly reported, with some recent post‐mortem studies reporting values ranging from 12 for a similar voxel size using a quadrature coil [Dell'acqua et al., 2013] to 65 in a 100 μm hippocampal sections at 80 × 80 × 50 μm voxel resolution using a microsurface coil [Portnoy et al., 2013]. It is know that fixation and time affect the water signal leading to a decrease in SNR [D'Arceuil and de Crespigny, 2007], as well as diffusion properties in post‐mortem samples [Richardson et al., 2014]. Indeed, decay of tissue quality is also a major issue in the procurement of appropriate human hippocampus control tissue, as CA1 pyramidal cells die within 15 minutes. Yet, for most available post‐mortem tissue, it takes at least 12 h before being fixed. The time to tissue fixation was minimal here (i.e. a few minutes) with a biopsy sample and time to scanning was also kept at <1 month postfixation to minimize these potential effects.

To determine the magnitude of signal contrast between different hippocampal layers, a straight line was drawn as a ROI across all layers and the signal intensity across the line was plotted. Drawing of ROIs at the 0.001 μL voxel size afforded a clear identification of different layers based on the signal intensity plots. A simple contrast ratio was subsequently calculated between the layers (e.g. DG/SM). Although this is akin to a contrast‐to‐noise ratio (CNR), none of the signal within these measurements is considered noise. It is hence more appropriate to refer to this measure as a simple contrast ratio that provides an indication of the magnitude of contrast required to reliably identify individual cell layers.

To calculate how voxel size affects SNR and layer detection, scans with a voxel size of 0.001 μL were used to resample images at different 3D voxel sizes of 0.008, 0.125, 1, and 8 μL in FIJI (version 2.0, NIH).

### Microtractography

For microtractography, a local multi‐direction deterministic fiber‐tracking algorithm for diffusion spectrum imaging [Yeh et al., 2013] was used for fiber reconstructions in DSI studio. It is important to note that due to the small voxel size here, partial volume effects that lead to multiple fiber directions and crossings are less of a concern in reconstruction approaches at the mesoscale [Bastiani et al., 2012]. For this, a mask was created to remove background and fiber directions were estimated for each voxel. No upsampling or motion correction was used in the processing. Tractographic reconstructions were based on the fractional anisotropy thresholded to 0.09 with an angular threshold of 60° (considered likely within grey matter), a step size of 0.1 mm (the physical size of an actual voxel edge), no smoothing, minimum length of 0.2 mm (i.e. connection of at least two voxel dimensions) and a maximum length of 100 mm. All seed orientations were considered with subvoxel seed positions and randomized sampling. All fibers passing through, originating or ending within the seed were considered. Direction interpolation was to the nearest point using a streamlined (Euler) tracking algorithm terminated after 1,000,000 random seeds for the whole sample (compared with 1,579,200 voxels for total sample). Tractograms were overlaid on an ADC map.

The reproducibility of the tractograms was determined by reprocessing 10 times the data from the original acquisition file. The total number of streamlines from a seed (100,000) in the dentate gyrus was measured to calculate an intra‐subject coefficient variation (CoV) [Besseling et al., 2012; Smith et al., 2015]. For this, streamlines for each re‐processed image were quantified to produce a mean number of streamlines (*μ =* 710) as well as a standard deviation (*σ* = 13.4). A division of the standard deviation by the mean produced a CoV of 0.019, indicating little variation between tractograms on the same subject.

### Connectivity Matrix

Regional fiber connectivity was calculated using DSI studio based on the fiber streamlines that pass through or terminate from one ROI to another. Each ROI is used to determine which streamlines from the other region pass through or end within a given ROI, hence counts using ROI 1 as seed are not necessarily the same than those from RO1 2 [Zalesky and Fornito, 2009]. The matrix graphically represents counts of each ROI being used as a seed. No fiber length normalization was used.

### Histological Processing and Immunohistochemistry

After MRI scanning was completed, the specimen was cryoprotected in 30% sucrose with 0.5% sodium azide before cutting 50 μm sections on a cryostat (Leica) directly onto microscopic slides. Sections were stored at −20°C. For immunohistochemistry, sections were washed 3 × 5 min in PBS before the overnight application of the primary antibodies. Primary antibodies consisted of the pan‐neuronal rabbit anti‐Fox3 antibody (1:1,000, Abcam, ab104225), the astrocytic marker mouse anti‐glial fibrillary acid protein (GFAP, 1:3,000 Sigma, G3893) and chicken anti‐myelin basic protein antibody (MBP, 1:100, Abcam, ab134018). The following morning, primary antibodies were removed and sections were washed 3 × 5 min with PBS before incubation with appropriate AlexaFluor secondary antibodies (1:500, Molecular Probes) for 1hr at room temperature and washed 3 × 5 min in PBS. The nuclear counterstain DAPI was then applied for 5 min at 1:10,000 before another 2 × 5 min washes in PBS, followed by a final wash in filtered dH_2_O and coverslipping with Vectashield for fluorescence (Vector Labs). Using an AxioImager M2 microscope (Zeiss) interfaced with a motorized stage controlled by Stereo Investigator software (MBF), individual multicolor microscopic images (×10 objective) were acquired before automatic tiling of these to reconstruct the entire section. A background removal function was run in FIJI to account for autofluorescence and inhomogeneities evident due to image tiling.

### Alignment of Histological and MR Images

Histological images were aligned to individual MR images using a landmark‐based registration function in FIJI. A total of 12 landmarks were used with six delineating edges of tissue and six defining within sample landmarks. Within sample landmarks were chosen based on ready identification within histological and MR images (e.g. edge of DG, middle of CA1). Although we previously described an efficient semi‐automated volumetric registration between histology and MR images [Stille et al., 2013], an individual section, rather than volumetric, registration was necessitated by the peripheral damage caused by cutting this fragile sample. Embedding of the whole sample in cryoprotective material can reduce these artifacts and enable a volumetric approach [Dahele et al., 2008]. Alternatively, tissue‐clearing methods can be applied to perform whole sample histology [Miyawaki, 2015], but this will limit the number of immunohistochemical markers that can be used for each sample.

## RESULTS

### Magnetic Resonance (MR)‐Histology of the Excised Human Hippocampus

Ex vivo high‐resolution multi‐parametric MR imaging affords the en bloc imaging of whole excised specimens using different image contrast mechanisms. Apparent diffusion coefficient (ADC) contrast maps are especially useful, providing detailed anatomical views of hippocampal architecture, including not only hippocampal layers, but also disease‐ or surgically‐induced pathology (Fig. 1D). Many of the features visible on the ADC map show correspondence to histological hallmarks, such as the dentate gyrus, detectable through immunohistochemistry in a single section cut along the same direction as the slices presented on the MR images (Fig. 1E). Indeed, an overlay of the merged immunohistochemistry image with the corresponding MR image illustrates how well the two data sets complement each other (Fig. 1F). Nevertheless, it is also evident that during sectioning some tissue elements were lost that were clearly present on the MR images. Indeed, this is one of the advantages of the ex vivo MRI scanning, it completely preserves the sample morphology without causing freezing or cutting artifacts. Mesoscopic features on the ADC map reveal subtle differences in intrahippocampal layers that afford a distinction between the dentate gyrus (DG) and CA4, but also between DG and stratum moleculare (SM) and stratum radiatum (SR) (Fig. 1G). Even a subtle intensity difference between SM and SR affords a distinction between these two layers that is not easily detected histologically based purely on cell density. These layers’ diffusion characteristics are also quite distinct from the denser CA1/2 layer that is broader than the DG in this particular location. Immunohistochemistry supports these MR histology features, but is still essential for optimal interpretation.

**Figure 2:**
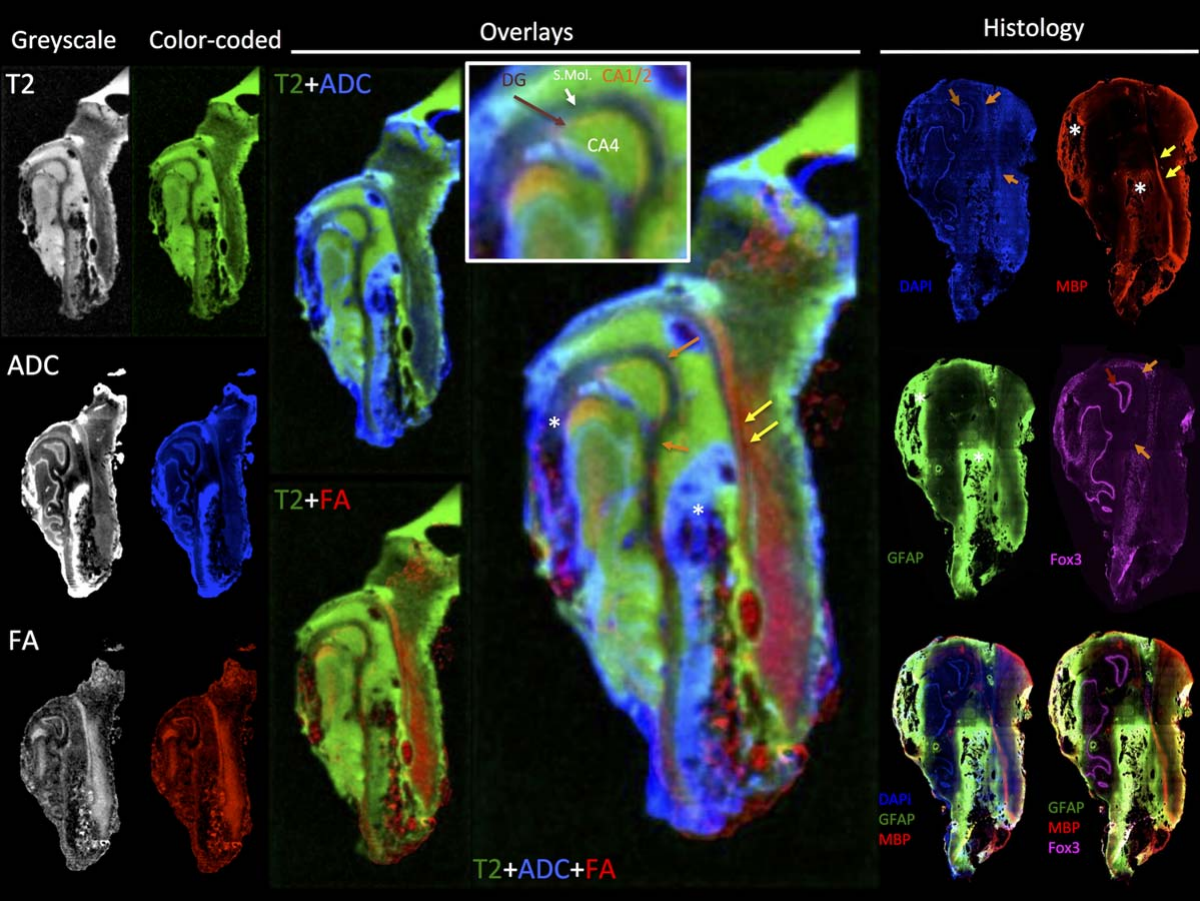
Diffusion‐based MR‐histology. A color‐coded synthesized MR‐histology (T_2_, ADC, FA) highlights histological features, such as the white matter (yellow arrows), DG (brown arrows) and CA1 cell layers (orange arrows), as well as tissue damage (white *). Myelinaed fibers (yellow arrows) of the subiculum are also evident. The tissue characteristics are also evident on fluorescent histology (DAPI, GFAP, MBP, FOX3). [Color figure can be viewed in the online issue, which is available at http://wileyonlinelibrary.com.]

**Figure 3:**
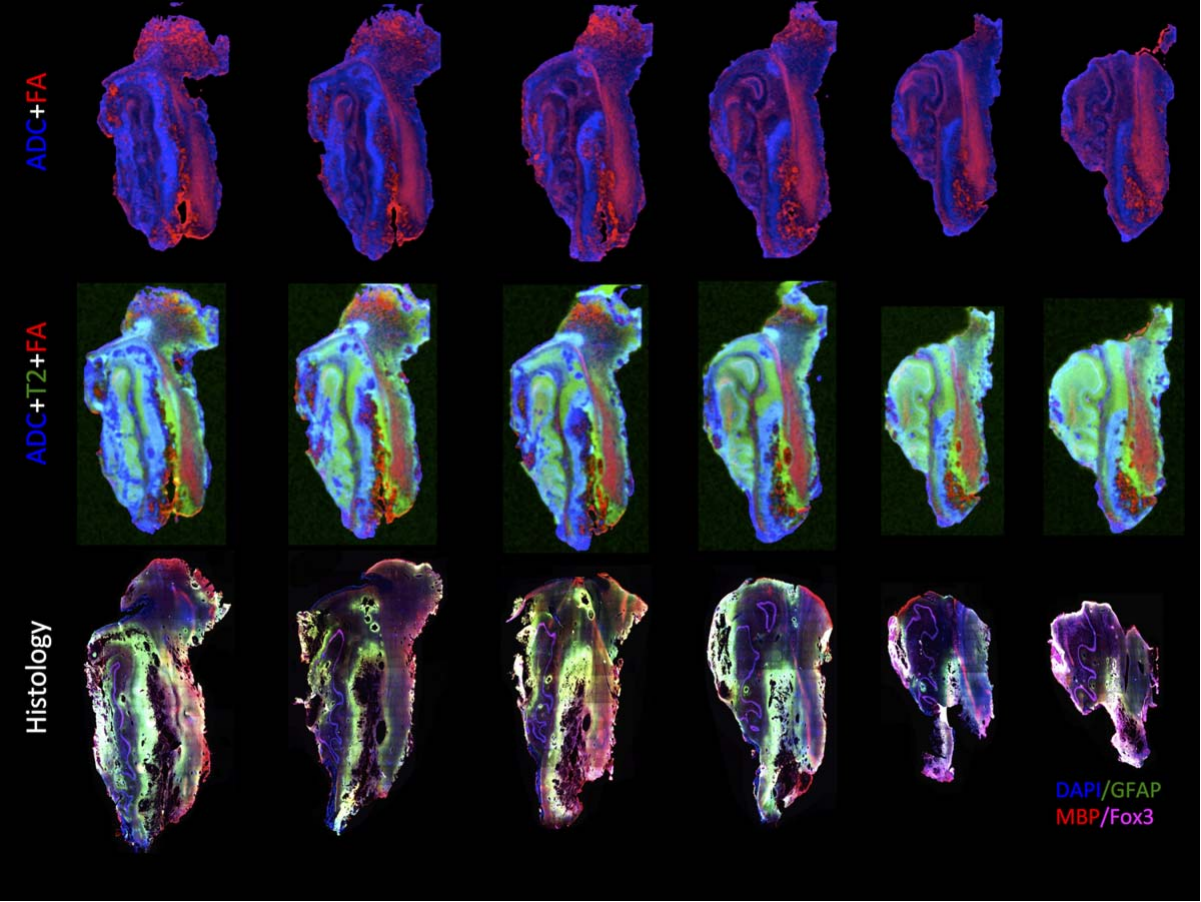
Comparison of MR histology with immunohistochemistry. A multislice overview of MR histology (T_2_, ADC, FA) and its correspondence to post‐mortem fluorescent histology (DAPI, GFAP, MBP, FOX3). It is evident that MR histology provides a similar mesoscale assessment of the hippocampus to histology, but does not suffer from potential cutting artifacts and hence a more complete view of the sample in 3D is available. Nevertheless, histology is required to provide a more detailed molecular characterization of anatomical features. [Color figure can be viewed in the online issue, which is available at http://wileyonlinelibrary.com.]

The richness of multiparametric MR data can be color‐encoded and integrated to yield overlay images that are akin to those generated by multiple markers using immunohistochemistry (Fig. 2). Specifically, T_2_‐weighted, ADC and fractional anisotropy (FA) maps can be combined, as they detect different aspects of tissue architecture, as evidenced by the overlays. This approach nicely delineates the different hippocampal layers. It is noteworthy that the T_2_‐weighted images by themselves do not readily identify these different layers, with only the alvear path exhibiting a hypointensity compared with the rest of the hippocampus proper. Importantly, the overlay of the MR images in 3D provides a clearer overview of the specimen than individual histology slices, especially areas of pathology and border regions in histological sections that are compromised compared with the MR dataset (Fig. 3).

### Delineating Hippocampal Subfields

**Figure 4:**
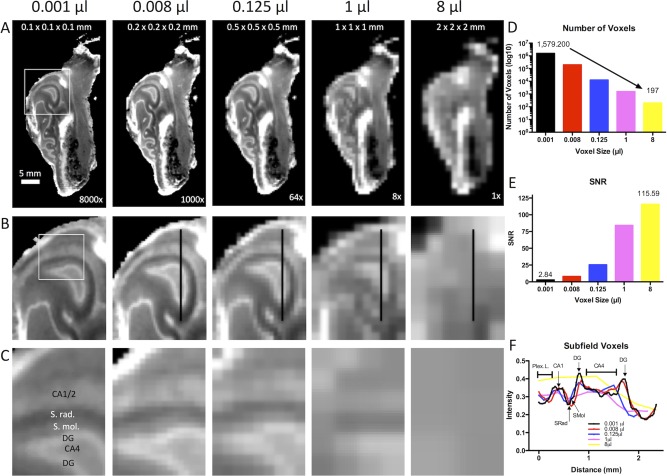
Partial volume effects. **A**. Voxel size for microtractography is 8,000× smaller than standard clinical protocols. An increased spatial resolution reduces partial volume effect and affords the discrimination of more detailed anatomical structures. **B**. For instance, a resolution of at least 1μL is required to define the dentate gyrus. **C.** Subfields of the hippocampus are only clearly discernable at a 0.125 μL volume resolution. **D**. The decrease in resolution also translates into a significantly larger number of voxels covering the excised samples compared with a comparable clinical resolution, dramatically increasing the overall information available from the sample. **E.** Nevertheless, a challenge to increase spatial resolution is the linear loss in signal‐to‐noise that requires adjustments in the acquisition strategy, such as higher field strength and increasing the number of averages. **F**. The magnitude of signal intensity change between subfields of the hippocampus is voxel size dependent, with higher resolutions not only providing sharper boundaries between fields, but also increasing the contrast between these layers. Hence, the smallest voxel size of 0.001 μL boast the greatest difference between signal intensity in the DG and stratum moleculare/radiatum (a ratio of 1.6) compared with the largest voxel size where this discrimination is possible (1.4). [Color figure can be viewed in the online issue, which is available at http://wileyonlinelibrary.com.]

The delineation of intrahippocampal structures is not only sequence‐dependent, but also requires the appropriate resolution to avoid partial volume effects (Fig. 4A). Partial volume effects of intrahippocampal structures can occur when these anatomical features are small in comparison with the larger voxel size within which these are contained. Essentially the signal of features is averaged or blurred out and prevents proper discrimination. Indeed, a resolution of at least 1 μL is necessary to afford the detection of intrahippocampal structures on an ADC map to be clearly discernable (Fig. 1B). A distinction of layers requires a resolution of at least 0.125 μL, but it is only at a 0.001 μL resolution where a clear separation can be seen. For intrahippocampal microtractography, it is hence essential to achieve a high resolution to deliver a clear separation of layers, each containing multiple voxels, for use as seed regions in microtractography. There is a substantial (×8,000) difference in voxels between 0.001 and 8 μL resolution (Fig. 4D) affecting acquisition time, data handling, as well as SNR (Fig. 4E). The trade‐off between spatial resolution and SNR therefore needs to be considered in conjunction with the biological information that is required to answer a particular question. Although SNR is higher at larger voxel sizes, this compromises the differentiation of different hippocampal layers based on signal intensity (Fig. 4F). To merely detect the DG and CA1/2 layer, a resolution of 1 μL is sufficient with a high SNR, in contrast increasing the spatial resolution to a degree where a distinction of SM and SR is possible comes at the cost of reducing SNR and incurring a severe penalty on acquisition time. We here see a DG/CA1 contrast ratio of 1.2, a DG/SM ratio of 1.68, and a CA1/S.R. ratio of 1.4. With increasing voxel size and hence a more pronounced partial volume effect, this ratio navigates gradually towards 1, although SNR increases. The delineation of intrahippocampal layers is hence less dependent on a high SNR, but requires sufficient contrast (i.e. signal intensity differences) between layers that is susceptible to partial volume effects as voxel size increases.

**Figure 5:**
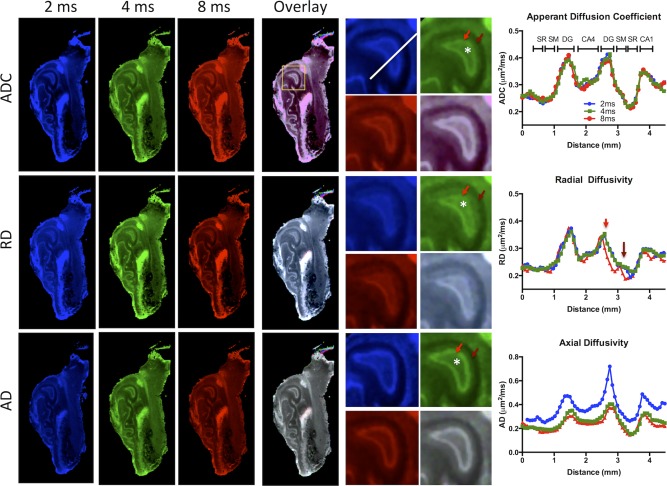
Diffusion duration and hippocampal subfields. At a 0.001 μL resolution, the intra‐voxel diffusion duration of water will be significantly different from the larger voxel sizes (8 μL) used clinically to reveal macroscopic white matter tracts. Diffusion duration of 2 (pseudo‐colored blue), 4 (green), and 8 ms (red) were therefore compared to investigate their influence on discerning hippocampal subfields using the apparent diffusion coefficient (ADC), radial diffusion (RD), or axial diffusion (AD). ADC and AD were able to discriminate CA4 (white *), dentate gyrus (red arrow) and stratum moleculare (brown arrow) for all diffusion duration. RD afforded some discrimination, but layer boundaries were not as clearly defined. A quantification of the diffusion signal along a defined line (white) across these subfields revealed that ADC provided a robust discrimination of layers across all diffusion duration. Interestingly, the 8 ms RD condition provide the sharpest boundary between stratum moleculare (SM) and radiatum (SR), but less so between SM and DG. For AD, 2 ms provided a distinctive signal that contrasted most clearly the DG from other layers. The acquisition of multiple diffusion durations and the potential combination of pseudo‐colored images therefore potentially offers new opportunities to more reliably distinguish different cell layers anatomically and to use this information to define regions of interests that can be used as seeds for intrahippocampal microtractography. [Color figure can be viewed in the online issue, which is available at http://wileyonlinelibrary.com.]

ADC provides the most robust image contrast for delineating different intrahippocampal structures compared with radial (RD) or axial diffusivity (Fig. 5). Different diffusion durations (2, 4, and 8 ms) were applied to determine their ability to delineate thin cellular layers, such as the dentate gyrus, versus more diffuse sparser seeded layers, such as SM and SR. However, overall there was little difference between these diffusion durations. Sharper differences between DG and CA4 could be detected on RD compared with ADC, but the DG layer itself was not as clearly defined. The longer 8 ms radial diffusion duration provided a better definition between SM and SR. Axial diffusivity in contrast was excellent at defining DG and CA1 layers in terms of width, but the magnitude of diffusion difference between layers was lowest, unless a long diffusion duration was applied. Specific intrahippocampal architecture at the mesoscale can hence be highlighted using different aspects and durations of diffusion. Pseudo‐coloring potentially affords the use of a multiparametric approach to synthesize images with maximum contrast between different layers.

### Microtractography of Extra‐ and Intrahippocampal Connectivity

**Figure 6:**
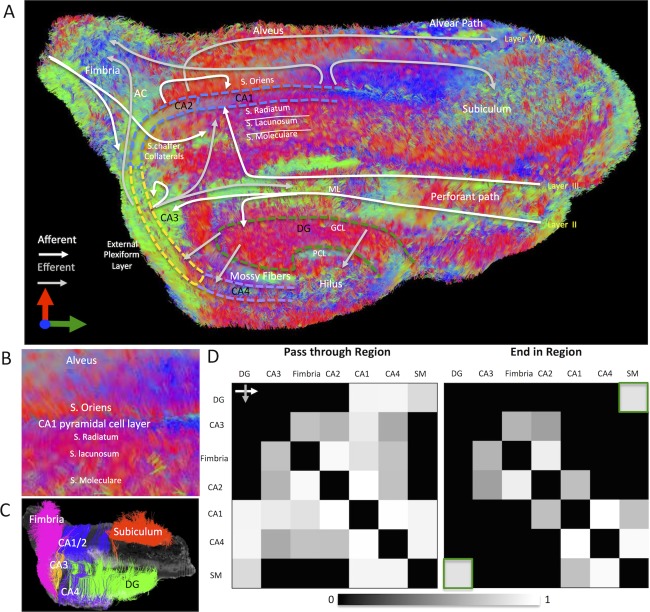
Connectivity matrix. **A**. Using 1,000,000 seeds randomly across the whole specimen, 541,527 tracts were found spanning at least two voxels (i.e. 200 μm) indicating a high connectivity with a mean tract length of 1.49 mm. This connectivity includes connections between layers, neighboring structures, but also tracts that connect regions with more remote areas. Most connections are short distance (<1 mm). Regional connectivity mapped onto a microtractography image displayed with directional color‐coding of all tracked fibers within the sample. The surface view of the edge where the sample has been cut in half reveals key neuroanatomical structures (CA1, CA2, CA3, CA4, DG), as well as the Fimbria and Subiculum where the perforant path enters the hippocampal structure. A schematic overlay of hippocampal connectivity indicates which areas are connected using afferent and efferent fibers. Note that part of the sample containing DG is missing due to the surgical resection. (AC = associational commissural pathway; ML = molecular layer; GCL = granule cell layer; PCL = polymorphic cell layer). Color‐coding indicates directionality of fiber orientations. **B**. Fiber characteristics afford a distinction of different hippocampal layers, such as the pyramidal cell layer of CA1, the overlying stratum oriens and the major efferent alvear path, but also stratum radiatum, stratum lacunosum and stratum moleculare. Color‐coding indicates directionality of fiber orientations, as in A. **C**. These definitions of neuroanatomical structure afford a systems view of hippocampal connectivity and confirm the assignment of neuroanatomical regions with, for instance, fibers from CA3 emanating into the fimbria, as well as CA1/2 and CA4. Each neuroanatomical region here uses different color to afford a visualization of fibers passing through this region. **D**. Connectivity matrices between these different pairs of neuroanatomical regions further provide a systems view that contrasts the tracts from each seed versus the number of streamlines that pass through or end within the region. However, these connectivity measures do not imply directionality, nor do they account for short fibers that provide intra‐regional connectivity. All connectivity strengths are normalized to the total number of tracts within the comparison and displayed on a grey scale between 0 (no connectivity) and 1 (highest connectivity). [Color figure can be viewed in the online issue, which is available at http://wileyonlinelibrary.com.]

Microtractography at a 100 μm isotropic resolution afforded the detection of extra‐ and intrahippocampal connections within the excised sample. To probe hippocampal connectivity, whole sample tractography was performed and displayed as streamlines color‐coded for directionality (Fig. 6A, Supporting Information 1). Anatomical regions were identified based on their specific tractographic characteristics and a schematic representing the functional anatomy was drawn to guide the placement of seeds. The pyramidal layers were readily identifiable based on diffusivity being mostly horizontal along the tissue slice, whereas ascending and descending fibers of the CA1 layer, for instance, revealing vertical streamlines (Fig. 6B). Streamlines further afforded a distinction of adjacent layers, such as the stratum oriens and radiatum. Based on these anatomical characteristics streamlines for each region were generated to reveal connectivity. The tractograms also help in identifying anatomical regions based on their known connectivity, for instance layer CA3 connecting with both CA1 and CA4, as well as the projecting fibers into the fimbria as part of the associational commissural pathway (Fig. 6C). A connectivity matrix between these different regions of interest revealed a high connectivity between a select number of anatomical regions (Fig. 6D). The mirror image of the connectivity matrix further reveal that equivalent numbers of streamlines are identified between two ROIs indicating that the location of the original seed does not affect the visualization of the connectivity between these two anatomical regions. It is noteworthy though that by assessing the end of streamlines in different ROIs, connections from the DG are terminating in the SM (or vice versa). These are thought to be aberrant connections and visualization using the streamlines will determine their precise location.

**Figure 7:**
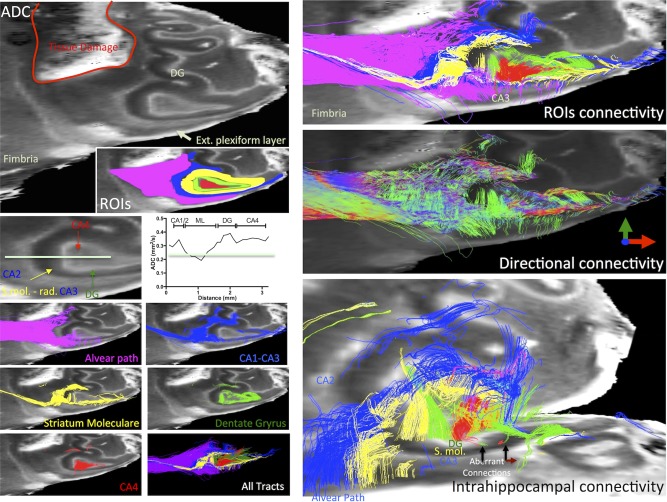
Intrahippocampal connectivity. Hippocampal cell layers are readily distinguishable on the ADC image and allow definition of individual ROIs that serve as seeds to probe passing fibers. Individual seeds defining the alvear path, CA1/2 layer, stratum moleculare/radiatum (SM), dentate gyrus (DG), and CA4 revealed intrahippocampal connectivity with some crossing of fibers between different ROIs. It was also evident that some fibers emanating from the DG projected to the SM (black arrow), while others appear to project to CA1 and beyond (brown arrow). This microtractography provides a unique view of intra‐ and extrahippocampal connectivity that is not easily achieved with histological investigations of individual sections. [Color figure can be viewed in the online issue, which is available at http://wileyonlinelibrary.com.]

To probe intrahippocampal connections further, select hippocampal layers were delineated as individual seeds to map tracts that pass through the anatomical structure, i.e. efferents and afferents (Fig. 7). As expected from histological studies, CA1/2 showed short connections with the stratum oriens throughout, but also connected out through the fimbria to the mammillary bodies and the alvear path to the cortex (mean length 2.61 mm). The SM/SR also project to the cortex through the alvear path and reveal short‐range connectivity with CA1/2 and the dentate gyrus (mean length 2.43 mm). The dentate gyrus mostly connects to CA3 and CA4 (mean length 2.44 mm). However, aberrant connections with the SM/SR are also evident, as well as a few connections with CA1/2. CA4 is mostly connected with the DG, but also CA3 (mean length 3.15 mm). This approach demonstrates for the first time that intrahippocampal connectivity can be probed using microtractography and that it is appropriate to detect putative aberrant connections that can potentially contribute to our understanding of circuits underlying epileptic activity.

## DISCUSSION

A major limitation to our understanding of the systems sub‐serving treatment‐resistant epilepsy is our inability to visualize extra‐ and intrahippocampal connections at the mesoscale [Bertram, 2009]. We here demonstrated that the surgically‐excised hippocampus can be noninvasively imaged in three dimensions at the mesoscale using MRI to create histology‐like images of tissue architecture with the added advantage of being able to visualize extra‐ and intrahippocampal connectivity. This methodological advance will provide a better characterization of the excised hippocampal tissue and afford an investigation of putative connectivity changes that could explain intractable epilepsy.

### Importance of the Mesoscale to our Understanding of Epilepsy

The hippocampus is an extensively studied neuroanatomical structure [Strange et al., 2014] with a pivotal role in treatment‐resistant epilepsy [Blumenfeld, 2014]. Characterizations of neuroanatomical changes due to or potentially causing treatment‐resistant epilepsy uncovered well‐documented macroscopic changes (e.g. volumetric loss, hypometabolism, etc.) as depicted by non‐invasive imaging methods, such as MRI and PET, as well as microscopic changes (e.g. diffuse CA1 layer, mossy fiber sprouting) revealed by histopathology. However, the mesoscale that encompasses intraregional connections remains mostly elusive [Bertram, 2009]. Yet, it is this intra‐regional connectivity that subserves potentially an epileptic network [Thom et al., 2010]. To create an integrated neurobiological framework to understand the many scales involved in epilepsy, the development of adequate imaging techniques at the mesoscale are required.

Dell'acqua et al. (2013) described the use of ex vivo MR histology and microtractography at the mesoscale to potentially inform this investigational gap. Using this approach, we here demonstrate that it is possible to determine an appropriate spatial resolution to visualize subfields of the hippocampus which is increasingly becoming a focus for clinical investigations [Goubran et al., 2014; Pipitone et al., 2014; Schoene‐Bake et al., 2014b; Thomas et al., 2008; Winterburn et al., 2013; Wisse et al., 2012]. It is evident here that substructures of the hippocampus and CA layers need a spatial resolution of at least 0.125 μL which is not achieved in many in vivo studies at clinical field strength. Indeed, it is likely that the plexiform layer that has a distinct MR signal compared with the rest of the hippocampus is misidentified as the CA1/2 subfield [Eriksson et al., 2009a]. An advantage of these high‐resolution ex vivo studies is that they also afford a validation by histology [Adler et al., 2014; Augustinack et al., 2013], ideally immunohistochemistry to relate MR features with specific anatomical markers. These ex vivo validation studies can provide the basis for novel sequences that can highlight specific hippocampal features and define an appropriate resolution to achieve their discrimination from other anatomical features. Indeed, we here demonstrate that the use of different MR parameters, such as ADC, T_2_ and FA, can be used to create MR histology images that highlight different anatomical features akin to immunohistochemistry [Dell'acqua et al., 2013].

However, the discrimination of different anatomical features from these images could also be influenced by the presence of subtle distortions or imaging‐related artifacts that, for instance, are related to gradient amplitude [Drobnjak et al., 2010]. Here a significant difference in diffusion gradient amplitude (342 vs. 867 mT/m) could lead to a gradient non‐linearity and eddy‐currents that would be least apparent at longest delta, hence resulting in sharper borders on AD maps acquired with a large delta. In contrast, in shorter delta AD maps subtle signal distortions could occur that lead to a blurring of signal boundaries. Sharp boundaries between anatomical features might hence also be influenced by the absence of subtle image distortions, rather than merely being a function of the type of image that is being generated. Further improvements in delineating anatomical features of the hippocampus can hence potentially be achieved by improving image acquisition. Investigating temporal scaling characteristics of the diffusion signal by using diffusion times could indeed lead to a more robust delineation of different structures [Özarslan et al., 2013]. For instance, Özarslan et al. have demonstrated a clear delineation of hippocampal subfields in the rat using this approach [Özarslan et al., 2012]. Application of these insights to a clinical environment can potentially afford an assessment beyond gross hippocampal atrophy, as well as allow measurements of validated subfield changes and their relationship to overall atrophy [Keller et al., 2012].

The correct identification of hippocampal anatomy is essential to conduct tractographical reconstructions exploring extra‐ and intrahippocampal connectivity. Several studies have investigated the anisotropic characteristics of human hippocampi ex vivo [Shepherd et al., 2007], including visualization of the perforant path [Augustinack et al., 2010]. However, microtractography of intrahippocampal connections requires a high isotropic spatial resolution to ensure that even small axonal projections can be traced between, for instance, the dentate gyrus and stratum moleculare (∼500–750 μm distance). The 100 μm isotropic resolution used here afforded the visualization of these 3D intrahippocampal connections. It is hence possible to probe if certain connections have been disrupted, but it is also an opportunity to evaluate hypotheses about potentially aberrant connections. Specifically, we were here able to demonstrate that, in this patient, there were a series of aberrant connections between the dentate gyrus and the stratum moleculare/radiatum and beyond, potentially indicating the role of CA1 damage in guiding the formation of emerging connections. Indeed, based on histological findings of inappropriate glutamatergic synapses in the stratum moleculare [Al Sufiani and Ang, 2012; Blumcke et al., 2012; Proper et al., 2000], it has been widely hypothesized that aberrant mossy fiber sprouting could subserve a reverberant excitatory network that cannot be controlled pharmacologically [Bertram, 2009]. However, due to a lack of appropriate methodology it was previously not possible to demonstrate this aberrant connection.

### Advancing Imaging Technology to Address Clinical Need

Demonstrating an aberrant connection between the dentate gyrus and stratum moleculare here is important to support the sprouting hypothesis and, specifically, the emergence of a reverberant excitatory network, but it raises a set of novel questions that require consideration if this technology is to eventually provide improved identification of pathological substrates that may be targets for novel therapeutic intervention in patients with intractable epilepsy. Specifically, it is unclear if aberrant connections are sufficient or necessary causes of intractable epilepsy. This raises the question of how many axonal connections may be required to create a reverberant network that is sufficient to sustain a seizure and whether the location of an aberrant bundle is a necessary condition [Ji et al., 2013] or whether these connections also might be found in non‐epileptic specimens. It was evident in this case that the aberrant connection was limited to a small region in the posterior part of the hippocampus. It is possible that other bundles or small connections between other regions are present, but were not easily detected using the described methodology. We can also not exclude the possibility that other uncharacteristic connections are present. Indeed, from a scientific and clinical point of view, it will not be possible to “visually” check each individual connection. A more automated system based on group class using deformation‐based morphometry that does not require white and grey matter classification is essential to provide a more robust analysis of aberrant connections [Afzali et al., 2011; Crum et al., 2013; Goubran et al., 2014; Pipitone et al., 2014].Group‐based image analysis at an appropriate resolution also needs to afford integration with measures of damage, such as a T1rho for gliosis or ADC/T2 overlays to visualize changes in CA fields in comparison to age‐matched controls, although some studies indicated no detection of neuronal loss and sclerosis using voxel‐based morphometry at the macroscale [Eriksson et al., 2009b]. Clinical histopathological analysis also fails to show significant changes in resected hippocampi in some cases [Usui et al., 2013].

A technical challenge hence presents itself in how one can address a clinical need using a methodology that relies on a high magnetic field strength and a spatial resolution 8,000× higher than what is currently available clinically. Ultra‐high field MRI with excellent spatial resolution is gradually finding its way into the clinic and increasingly specific diagnostic challenges are required to drive its implementation and development. Indeed, using a 7 T clinical MRI scanner combined with multi‐array coils, a resolution of 700 μm is achievable [Strotmann et al., 2014] and with postprocessing a “super‐resolution” of 200μm isotropic resolution becomes a realistic target in human subjects [Calamante et al., 2013]. Although the array of applications will remain far more limited than at lower field strengths, specific indications, such as uncovering aberrant hippocampal connections, might be on the clinical horizon pending further developments on ex vivo samples.

However, this technical prowess might not even be required. Using high resolution ex vivo resected epileptic hippocampi, it is possible to develop novel biological insights into the causes of aberrant connections, but this resolution and detail might not be a prerequisite at the preoperative diagnostic stage to advance the development of novel interventions. Most pharmacological therapies were indeed developed without being able to visualize specific receptors in patients, but the biological understanding was based on histopathology. In this case for instance, understanding where, and in which cases, aberrant connections develop, should inform our understanding of their structure‐function relationship and may clarify their potential as therapeutic targets in mesial temporal lobe epilepsy.

## CONCLUSION

An improved understanding of intractable epilepsy and the development of novel interventional approaches can hence be envisaged. For this, ideally in vivo high field MR (>7 T) imaging with ex vivo ultra high field (>11.7 T) MR imaging of excised specimen is combined with the immunohistochemical assessment of molecular and cellular pathology to validate the cytoarchitecture and connectivity attributed on MR [Howe et al., 2010]. These novel developments will provide a new direction to tackle intractable epilepsy.

## Supporting information

Supporting Information Movie 1.

Supporting Information Movie 2.
